# The Benefits to Bone Health in Children and Pre-School Children with Additional Exercise Interventions: A Systematic Review and Meta-Analysis

**DOI:** 10.3390/nu15010127

**Published:** 2022-12-27

**Authors:** Callum McCaskie, Aris Siafarikas, Jodie Cochrane Wilkie, Vanessa Sutton, Paola Chivers, Nicolas H. Hart, Myles C. Murphy

**Affiliations:** 1 School of Medical and Health Sciences, Edith Cowan University, Joondalup, WA 6027, Australia; 2 Department of Endocrinology and Diabetes, Perth Children’s Hospital, Nedlands, WA 6009, Australia; 3 School of Medicine and Telethon Kids Institute, University of Western Australia, Crawley, WA 6009, Australia; 4 Institute for Health Research, The University of Notre Dame Australia, Fremantle, WA 6160, Australia; 5 Western Australian Bone Research Collaboration, Fremantle, WA 6160, Australia; 6 Faculty of Health, Southern Cross University, Gold Coast, QLD 4225, Australia; 7 Nutrition and Health Innovation Research Institute, School of Medical and Health Sciences, Edith Cowan University, Joondalup, WA 6027, Australia; 8 Caring Futures Institute, College of Nursing and Health Science, Flinders University, Adelaide, SA 5042, Australia; 9 School of Sport, Exercise and Rehabilitation, Faculty of Health, University of Technology Sydney, Moore Park, NSW 2007, Australia; 10 School of Health Sciences and Physiotherapy, The University of Notre Dame Australia, Fremantle, WA 6160, Australia

**Keywords:** DXA, pQCT, impact exercise, jump

## Abstract

Objective: Determine if exercise interventions, beyond what is already provided to children and preschool children, improve bone health and reduce fracture incidence. Design: Systematic review and meta-analysis reported using the PRISMA guidelines. Certainty of evidence was assessed using GRADE recommendations. Data sources: Five electronic databases were searched for records: PUBMED; CINAHL; CENTRAL; SPORTDiscus; Web of Science. Eligibility criteria for selecting studies: Randomised, quasi-randomised and non-randomised controlled trials (including cluster-randomised) assessing the impact of additional exercise interventions (e.g., increased physical education classes or specific jumping programs) on bone health in children (6–12 years) and pre-school children (2–5 years) without dietary intervention. Results: Thirty-one records representing 16 distinct clinical trials were included. Dual-energy X-ray Absorptiometry (DXA) and/or peripheral Quantitative Computed Tomography (pQCT) were used to quantify bone health. Increased femoral neck bone mineral content in children with additional exercise interventions (*n* = 790, SMD = 0.55, 95% CI = 0.01 to 1.09) was reported, however this was not significant following sensitivity analysis. Other DXA and pQCT measures, as well as fracture incidence, did not appear to significantly differ over time between intervention and control groups. No studies reported adverse events. Studies failed to report all domains within the TIDieR checklist. All studies were at high risk of bias using the Cochrane RoB Tool 2.0. The certainty of the evidence was very low. Conclusions: The addition of exercise interventions, beyond what is provided to children, does not appear to improve DXA and pQCT measures of bone health. The effect of additional exercise interventions on bone health in pre-school children is largely unknown. Future trials should ensure adherence is clearly reported and controlled for within analysis as well as including reports of adverse events (e.g., apophysitis) that occur due to increased exercise interventions.

## 1. Introduction

It is widely agreed that bone mass accrual in childhood and adolescence determines peak bone mass and the risk of osteoporosis later in life [1,2]. Furthermore, the accrual of bone mass can be optimised by mechanical loading through exercise interventions [3,4,5,6] with bone adaptation most readily occurring with mechanotransduction of impact loading (e.g., running or jumping) [7,8]. Engagement in activities involving running and jumping is not only beneficial for bone, but also has wide ranging health benefits, including reducing obesity and improving psychological well-being [5].

The World Health Organisation recommends “children and young people aged 5–17 years old should accumulate at least 60 min of moderate to vigorous-intensity physical activity daily” [9]. However, a recent analysis from the Australian Institute for Health and Welfare showed that less than one-quarter of children aged 5–14 years meet this recommendation [10]. Worryingly, fracture rates within Australia have also been shown to have increased over time [11].

Given the importance of specific exercise to bone development, such as running or jumping, one of the simplest solutions would be, in theory, to increase exercise in children to optimise benefits from exercise starting early in life. If being specific to bone health, the exercise interventions would need to include specific aspects such as timing and duration of exercise, motivation and mode of activity [12]. Whilst the effect of exercise interventions for bone health in children have been quantified previously, new research is available. Furthermore, these previous systematic reviews contain methodological concerns. Behringer et al. 2014 pooled the results of all studies, irrespective of the region Bone Mineral Content (BMC) or areal bone mineral density (aBMD) was assessed, at their final follow-up with these time points ranging from 6–208 weeks, which given the time taken for bone to adapt following mechanical loading is a problem. Ref. [3] Tan et al. 2014 listed studies that had assessed the same data at different timepoints separately, influencing conclusions, as opposed to treating all published papers related to a single cohort as one trial (e.g., Malmo Paediatric Osteoporosis Prevention Study). Ref. [6] Nikander et al. 2010 also failed to delineate multiple studies using the same data and included both in pooled analysis [13,14], and presented pooled results within meta-analysis of outcomes assessing different regions (e.g., tibia and femoral measures were pooled).

Due to the sample sizes of previous meta-analyses being incorrectly inflated due to the inclusion of several published studies from a single trial or by pooling different body regions, the positive relationship between existing exercise regimes and bone health shown in previous reviews remains uncertain.

### Objectives

Our primary objective was to determine if additional exercise interventions, beyond what is already provided, in children and preschool children improve bone health. Our secondary objective was to determine if additional exercise interventions, beyond what is already provided, in children and preschool children reduce fracture incidence.

## 2. Materials and Methods

### 2.1. Guidelines

The systematic review and meta-analysis was designed and reported in accordance with the Preferred Reporting Items for Systematic Reviews and Meta-Analyses (PRISMA) [15].

### 2.2. Prospective Registration

This systematic review protocol has been registered with PROSPERO (CRD42022300902) on the 14^th^ May 2022 [https://www.crd.york.ac.uk/prospero/].

### 2.3. Data Management

All records were stored online using Covidence (Covidence systematic review software, Veritas Health Innovation, Melbourne, Australia). All extracted data were stored in a Microsoft Excel document, within Microsoft Teams.

### 2.4. Inclusion Criteria

#### 2.4.1. Participants

We included children (6–12 years) and preschool children (2–5 years) within this review who did not have medical comorbidities. We did not restrict inclusion based on body mass index.

#### 2.4.2. Primary Outcomes

The primary outcome of interest for this review was bone health. We included any measure of bone assessed using Dual-energy X-ray Absorptiometry (DXA) or peripheral Quantitative Computed Tomography (pQCT) with baseline and follow-up outcomes.

#### 2.4.3. Secondary Outcome

We included the incidence of fractures (occurrences) as a secondary outcome of this systematic review.

#### 2.4.4. Types of Studies

We included randomised, quasi-randomised and non-randomised clinical trials where at least one study arm used an exercise intervention for bone health and was compared to a control group (standard care/standard physical activity or other exercise intervention). Observational trials, case reports or series, clinical observations and systematic reviews were excluded.

### 2.5. Search Strategy

A single study author (MM) performed all search strategies from database inception to 3 May 2022 and downloaded all records into Endnote 20.4.1 (Clarivate Analytics, Philadelphia, PA, USA), and subsequently uploaded them into Covidence.

#### 2.5.1. Electronic Searches

Searches using free-text terms (Appendix A) were performed within the following electronic databases; PUBMED, CINAHL (Full-text), CENTRAL, SPORTDiscus, Web of Science. Limitations were used, where able, within individual databases with the complete search documentation provided within Appendix B.

#### 2.5.2. Searching Other Sources

Reference lists of reviews and retrieved articles were checked for additional studies missed in the electronic database search. The ePublication lists of key journals in the field were screened to identify studies that had yet to be indexed.

#### 2.5.3. Study Selection

Titles and abstracts of records identified by the search strategy were screened for their eligibility by two independent reviewers (MM/CM). Full-text screening was performed by the same two independent reviewers (MM/CM). Disagreements were resolved via consensus. The overall reasons for full-text articles being excluded are reported within the PRISMA flow chart [15].

### 2.6. Dealing with Multiple Records from the Same Cohort

Where multiple records were identified for a single cohort (as identified directly via manuscript text, referencing, clinical trial registration matching or ethical approval number matching) records were pooled to represent a single trial. Nine records were identified from the Swedish Malmo Paediatric Osteoporosis Prevention study [16,17,18,19,20,21,22,23,24]. Two records were identified from the Canadian AS!BC study [13,14]. Three records were identified from the Canadian Impact Loading study [25,26,27]. Three records were identified from the American Jumping Intervention study [28,29,30]. Two records were identified from the Swiss jumping study [31,32].

### 2.7. Dealing with Missing Data

Where the baseline or follow-up mean was not reported, the corresponding authors were contacted to provide these data directly. Ten studies did not provide a follow-up mean within their manuscript and were contacted for these data [18,19,24,26,32,33,34,35,36,37]. The corresponding author of two studies reported the data no longer exists [33,34]. Eight study authors did not respond to a request for data [18,19,24,26,32,35,36,37], however this was not unexpected with many studies being greater than ten years old. Where a standard deviation was not reported, yet the confidence interval was, a standard deviation was obtained using the methods described within the Cochrane Handbook for Systematic Reviews of Interventions [38]. Where no measure of deviation was provided, the standard deviation was imputed from a trial with an identical outcome measure at the closest timepoint, as recommended within the Cochrane Handbook for Systematic Reviews of Interventions [38].

### 2.8. Data Extraction

Data were abstracted from studies into Microsoft Excel independently by two review authors (CM/VS). Disagreements were resolved via group consensus with a senior reviewer (MM). The following information was abstracted from included studies: primary author; year of publication; country of origin; funding; competing interests; study design; study population; overall sample size; age range; details related to the intervention to complete the Template for Intervention Description and Replication (TIDieR) [39] checklist; mean (SD) age; mean (SD) weight; mean (SD) height; mean (SD) body mass index; participant sex; sample size at baseline and follow-up, mean (SD) of outcome variable (e.g., BMC) at baseline and follow-up; number of fractures.

### 2.9. Assessment of Methodological Quality

Two independent review authors (CM/AS) assessed the quality of included studies using the Cochrane Risk of Bias 2.0 Tool [40]. Disagreements were resolved via group consensus with a senior reviewer (MM). An overall judgement of methodological quality was assigned based on a ‘worst-item-counts’ basis with studies being assigned ‘high risk’ if at least one item was reported as “high risk”, unclear if no items are reported as ‘high risk’ and at least one item is reported as ‘unclear risk’, and ‘low risk’ if all items are reported as ‘low risk’ [41,42].

### 2.10. Assessment of Diversity and Heterogeneity

A Chi square test was used to evaluate statistical heterogeneity [38]. The I^2^ statistic informed between study heterogeneity based on the *p* value being <0.10 or the I^2^ value being >40% [38].

### 2.11. Assessment of Reporting Biases

The influence of small sample and publication bias were considered. Publication bias was not assessed using Egger’s test [43] due to the small number of studies (<10) included within each meta-analysis [38]. Alternatively, funnel plots were inspected when there were greater than five studies included within meta-analysis. We used the risk of bias criterion ‘sample size’ to account for small study biases, as per previous meta-analysis [44]. Where fewer than 50 participants were included, the study was classified as high-risk. Where between 50 and 200 participants were included, studies were classified as moderate risk and when greater than 200 participants were included studies were classified as low risk of small sample bias [45,46].

### 2.12. Data Synthesis

Data abstracted from included studies are presented within our Summary of Findings Table. The standardised mean difference (95% confidence intervals) was determined from pooled studies using random-effects, inverse variance models within Review Manager. Statistical significance was identified at *p*  <  0.05.

### 2.13. Sensitivity and Subgroup Analysis

We had planned to perform sub-group analysis, however based on the limited number of studies we did not proceed with this. We performed sensitivity analysis for meta-analyses consisting of three or more studies and demonstrating statistical heterogeneity with a clear outlier. Sensitivity analysis was conducted by excluding the outlier and exploring whether that altered the significance of the meta-analysis. With the limited number of included studies, and the lack of statistical heterogeneity observed within most meta-analyses, sensitivity analysis was not performed for every meta-analysis.

### 2.14. Assessment of the Certainty of the Body of Evidence

We assessed the certainty of the body of evidence using the GRADE approach (as recommended in the Cochrane Handbook for Systematic Reviews of Interventions) [47]. This process involved overall judgements on the quality of the body of evidence based on the overall risk of bias, inconsistency, indirectness, imprecision and publication bias.

### 2.15. Deviations to Protocol

We had planned to extract components of included study interventions using the Consensus on Exercise Reporting Template (CERT) framework [48], however due to the broader nature of the interventions prescribed within included studies this was replaced with the TIDieR checklist.

## 3. Results

### 3.1. Selection of Studies

**Figure 1:**
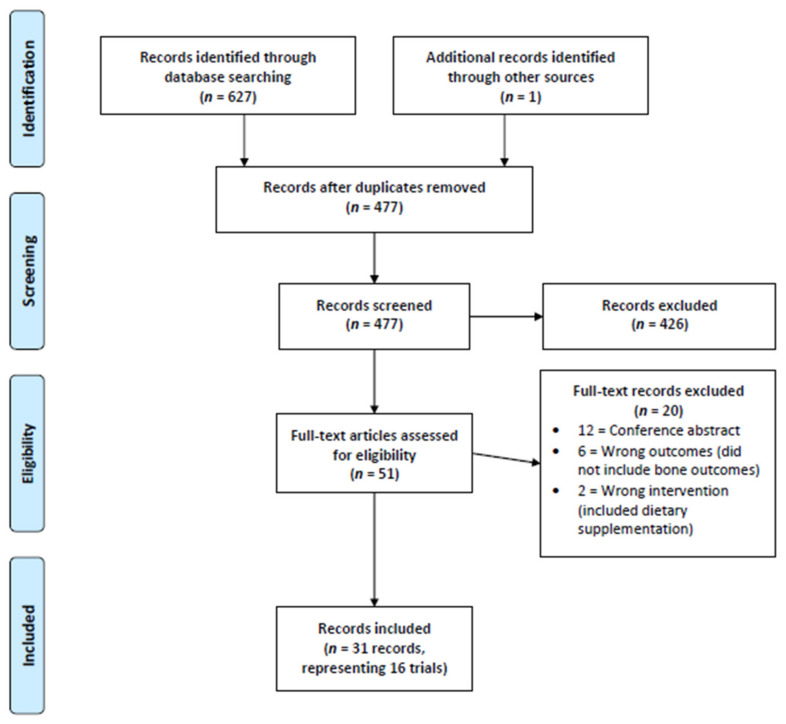
PRISMA Flow Chart.

We identified 477 records following the removal of duplicates within Covidence. After title and abstract screening, 51 records proceeding to full-text review with 31 records being included within this review [13,14,16,17,18,19,20,21,22,23,24,25,26,27,28,29,30,31,32,33,34,35,36,37,49,50,51,52,53,54,55]. These 31 records represented 16 distinct clinical trials (e.g., 15 studies were follow-up publications from the original study publication). Study inclusion is demonstrated within our PRISMA flow chart (Figure 1) and a study-by-study list of exclusion is provided within Appendix C.

### 3.2. Study Information

Full study data are presented within Table 1, with funding information presented within Appendix D. Of the sixteen distinct trials, 15 were randomised [13,16,25,28,32,34,35,36,37,49,50,51,53,54,55] and one was not [52]. Of the 15 randomised trials, four were individually randomised [28,36,37,50] and eleven were cluster randomised [13,16,25,30,32,35,49,51,53,54,55]. Fifteen trials included children [13,16,25,28,32,34,35,37,49,50,51,52,53,54,55] and one trial included pre-school children [36]. One trial exclusively included overweight or obese children [37]. Trials were performed in a variety of countries: Five in the United States of America [28,34,36,37,50]; three in Canada [13,25,53]; two in Australia [51,55]; two in Denmark [35,52]; two in Switzerland [32,49]; one in South Africa [54]; and one in Sweden [16].

### 3.3. Intervention Information

The TIDieR checklist was completed to detail all information related to the interventions provided within each included study, and is presented within Appendix E. All trials compared additional exercise interventions to standard exercise in children and pre-school children and most (11/16) trials used additional exercises specifically selected for bone adaptation, predominantly based around additional jumping exercise [13,16,25,28,32,35,36,37,49,53,55]. The duration of additional exercise interventions varied from 20 weeks to four years between studies, with all additional exercise interventions being performed face-to-face. Little equipment was needed for most studies and the interventions typically took place at school.

### 3.4. Effect of Exercise Interventions on Dual-Energy X-ray Absorptiometry Measures

The complete dataset for DXA outcomes are presented within Appendix F and all studies reported below assessed children, not pre-school aged children.

#### 3.4.1. Bone Mineral Content

**Figure 2:**
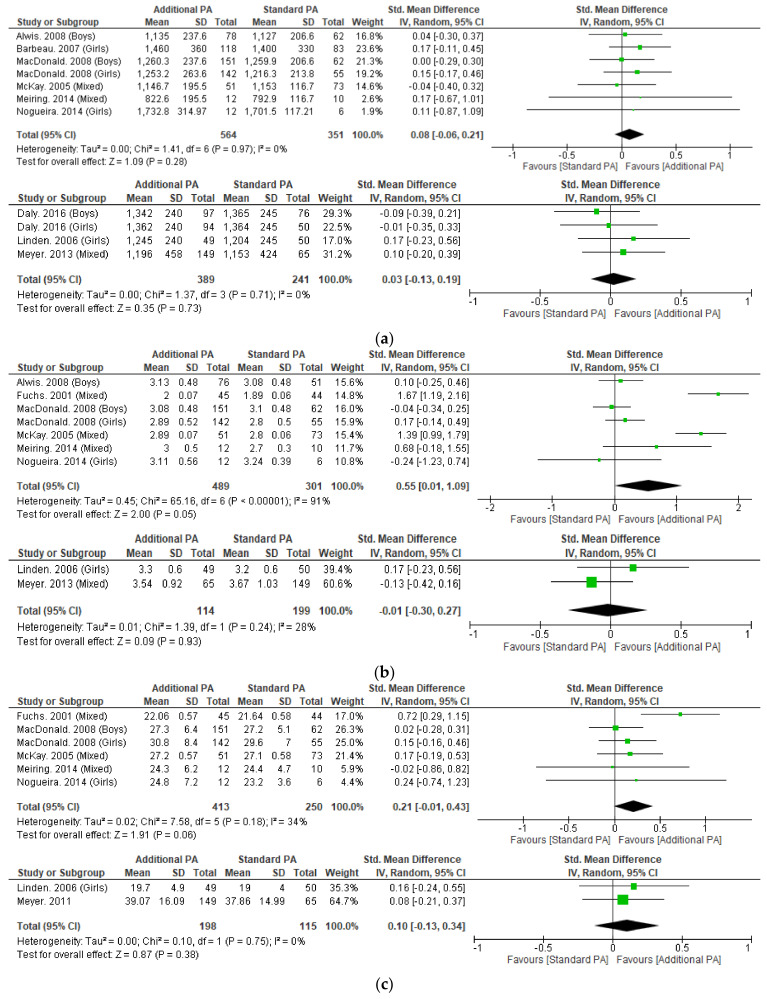
(**a**) Effect of additional exercise interventions on whole body bone mineral content. (**Top**) Less than 15 months follow-up, (**Bottom**) 24–48 months follow-up. Legend: PA = Physical Activity, Std = standardised, IV = inverse variance, SD- standard deviation, CI = confidence interval. (**b**) Effect of additional exercise interventions on femoral neck bone mineral content. (**Top**) Less than 15 months follow-up, (**Bottom**) 24–48 months follow-up. Legend: PA = Physical Activity, Std = standardised, IV = inverse variance, SD- standard deviation, CI = confidence interval. (**c**) Effect of additional exercise interventions on lumbar spine bone mineral content. (**Top**) Less than 15 months follow-up, (**Bottom**) 24–48 months follow-up. Legend: PA = Physical Activity, Std = standardised, IV = inverse variance, SD- standard deviation, CI = confidence interval.

Meta-analysis was possible for BMC of the whole body, femoral neck, and lumbar spine (Figure 2). Only short-term femoral neck BMC was significantly improved with additional exercise interventions, when compared to standard exercise (SMD = 0.55, 95% CI 0.01 to 1.09, *p* < 0.05). Sensitivity analysis performed on short-term femoral neck BMC by excluding Fuchs et al. 2001 as an outlier made this result non-significant. No other measure of BMC was significantly changed with additional exercise interventions.

#### 3.4.2. Areal Bone Mineral Density

**Figure 3:**
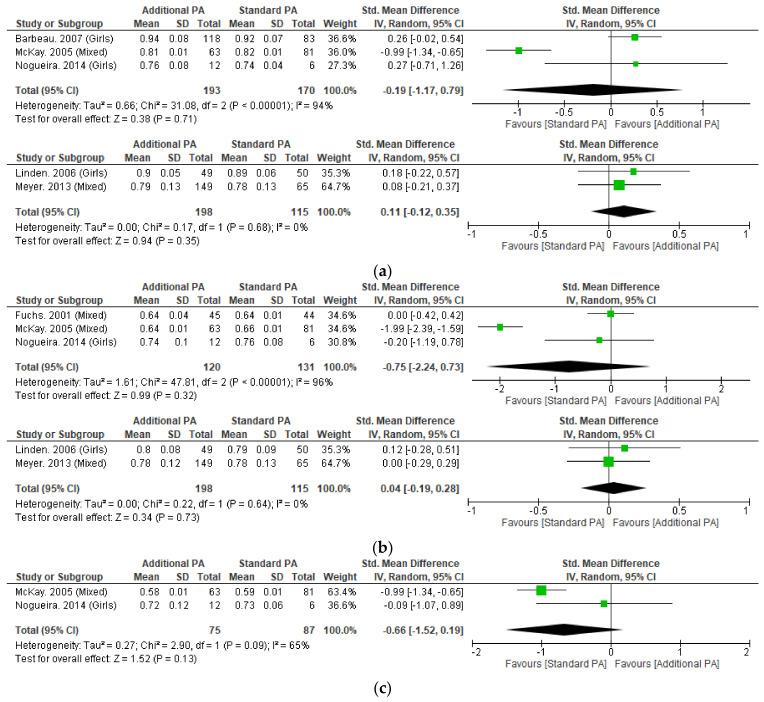
(**a**) Effect of additional exercise interventions on whole body areal bone mineral density. (**Top**) Less than 15 months follow-up, (**Bottom**) 24–48 months follow-up. Legend: PA = Physical Activity, Std = standardised, IV = inverse variance, SD- standard deviation, CI = confidence interval. (**b**) Effect of additional exercise interventions on femoral neck areal bone mineral density. (**Top**) Less than 15 months follow-up, (**Bottom**) 24–48 months follow-up. Legend: PA = Physical Activity, Std = standardised, IV = inverse variance, SD- standard deviation, CI = confidence interval. (**c**). Effect of additional exercise interventions on lumbar spine areal bone mineral density at less than 15 months follow-up. Legend: PA = Physical Activity, Std = standardised, IV = inverse variance, SD- standard deviation, CI = confidence interval.

Meta-analysis was possible for aBMD of the whole body, femoral neck and lumbar spine (Figure 3). No significant differences were detected with additional exercise interventions, when compared to standard exercise. Sensitivity analysis performed on short-term whole body and femoral neck aBMD by excluding McKay et al. 2005, which resolved heterogeneity and the results remained not significant.

#### 3.4.3. Bone Cross-Sectional Area

Meta-analysis was possible for cross-sectional area (CSA) of the femoral neck for outcomes less than 12 months (Appendix G). No significant differences were seen with additional exercise interventions, when compared to standard exercise at less than 12 months within the femoral neck. In additional to the meta-analysis, individual studies that measured CSA within the femoral neck at eight years and the lumbar spine at 6 months did not demonstrate significant differences between groups [16,17,28].

#### 3.4.4. Bone Area and Width

Meta-analysis was not possible for any measures of bone area or width, with no significant differences being reported within the study that assessed these measures [23,27].

### 3.5. Effect of Exercise Interventions on Peripheral Quantitative Computed Tomography Measures

The complete dataset for pQCT outcomes is presented within Appendix H and all studies reported below assessed children, not pre-school aged children.

#### 3.5.1. Volumetric Bone Mineral Content

Meta-analysis was possible for 4% tibial volumetric bone mineral content (vBMC) with no significant differences seen with additional exercise interventions, when compared to standard exercise (Appendix I). Meta-analysis was not possible for other measures of volumetric bone mineral content (vBMC), with no significant differences being reported within the one study that observed these measures at different sites of the tibia (14%, 38%, and 66%) [49].

#### 3.5.2. Volumetric Bone Mineral Density

**Figure 4:**
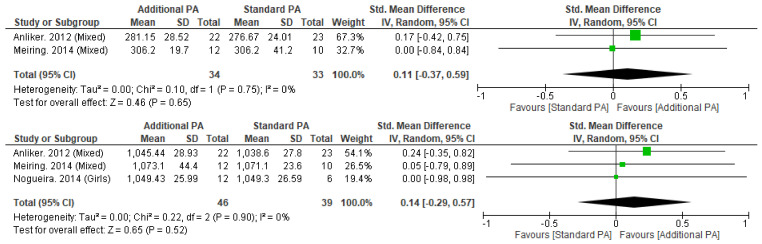
Effect of additional exercise interventions on volumetric bone mineral density at less than 12 months follow-up measures. (**Top**) 4% Tibia, (**Bottom**) 38% Tibia. Legend: PA = Physical Activity, Std = standardised, IV = inverse variance, SD- standard deviation, CI = confidence interval.

Meta-analysis was possible for tibial vBMD at 4% and 38% sites with no significant differences seen with additional exercise interventions, when compared to standard exercise (Figure 4). Individual studies of the tibia (4%, 8%, 14%, 35%, 50%, 66%) and radius (38%) also did not observe significant between-group differences [13,49,51,54,55].

#### 3.5.3. Trabecular Bone Mineral Density

Meta-analysis was possible for 4% tibial trabecular vBMD with no significant differences seen with additional exercise interventions, when compared to standard exercise (Appendix J). In additional to the meta-analysis, one study of the radius (4%) did not observe significant between-group differences [54,55].

#### 3.5.4. Bone Cross-Sectional Area

Meta-analysis was possible for tibial CSA at 4% and 38% sites with no significant differences seen with additional exercise interventions, when compared to standard exercise (Appendix K). Individual studies of the tibia (8%, 14%, 50%, 66%) did not observe significant between-group differences [13,49,51,54].

#### 3.5.5. Total Cortical Area

Meta-analysis was not possible for any measures of total cortical area of the tibia (38%, 50%, 66%), with no significant differences being observed within the studies that reported these measures [13,51,54].

#### 3.5.6. Total Cortical Thickness

Meta-analysis was not possible for any measures of total cortical thickness of the tibia (38%, 66%), with no significant differences being observed between studies that reported these measures [51,54].

#### 3.5.7. Stress–Strain Index

Meta-analysis was possible for 38% tibial polar stress–strain index (SSIPOL) with no significant differences seen with additional exercise interventions, when compared to standard exercise (Appendix L). Individual studies of the tibia (14%, 50%, 66%) did not observe significant differences [13,49,51,54].

#### 3.5.8. Bone Strength Index

Meta-analysis was not possible for the tibial bone strength index (4%, 8%), with no significant differences being observed within the studies that reported these measures differences [13,54].

#### 3.5.9. Other

Meta-analysis was not possible for tibial endosteal circumference or periosteal circumference (38%). No significant differences were observed within the studies that reported these measures [54,55].

### 3.6. Fracture Incidence

**Figure 5:**
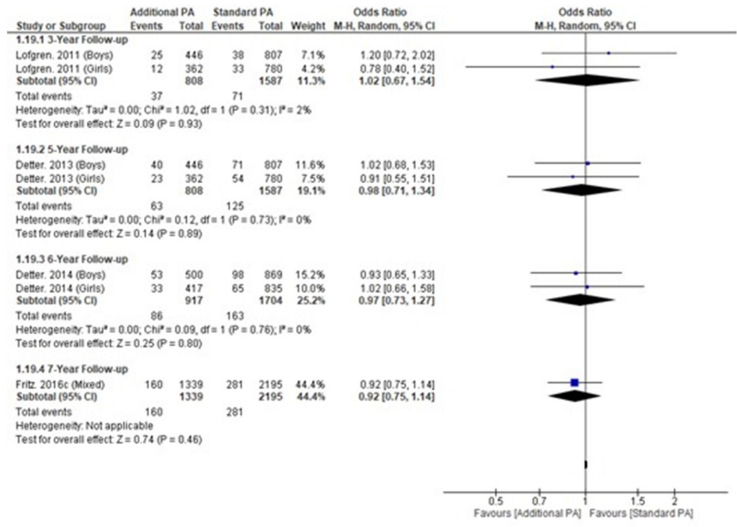
Fracture incidence within a single longitudinal trial over time.

Fracture incidence was reported in children, not preschool children, within a single trial, with four publications reporting incidence at different timepoints (Appendix M) and no significant differences at any follow-up time point (Figure 5) [18,19,20,24].

### 3.7. Assessment of Quality in Included Studies

All studies were judged to be at high-risk of bias with the overall risk of bias judgements presented within Appendix N. Risk of bias arising from the randomisation process was low in 88% of included studies. Risk of bias arising from the timing of identification or recruitment of participants into the randomised trial was low in all studies (100%). No studies reported a low risk of bias due to deviations from the intended interventions (effect of assignment to intervention). One study was judged as low risk (6%) for risk of bias due to deviations from the intended interventions (effect of adhering to intervention). Risk of bias due to missing outcome data was low for 94% of studies. All studies were low risk (100%) for risk of bias in measurement of the outcome and risk of bias in selection of the reported result. Finally, funnel plots were inspected for all meta-analyses with greater than five included studies and did not indicate any evidence of publication bias.

### 3.8. Assessment of the Certainty of the Body of Evidence

This systematic review and meta-analysis included randomised and non-randomised controlled clinical trials, with the initial level of evidence (before GRADE judgements) classified as high. Study limitations, from risk of bias, downgraded the certainty of the evidence once. As most meta-analyses reported the majority of individual study confidence intervals crossing zero, minimal heterogeneity was seen between studies, and if present could be resolved by the exclusion of a single study. Thus, the certainty was not downgraded for inconsistency. Trials included similar populations, interventions and outcome measures, so the certainty of the evidence was not downgraded for indirectness. Trials included samples unlikely to be large enough to detect between group effect sizes and the certainty of the evidence was downgraded two levels for imprecision. There was no publication bias detected via funnel plots and the certainty of the evidence was not downgraded for publication bias. The overall certainty of the evidence was judged to be very low, meaning little confidence exists in the reported effect sizes and that the true effects are likely to be substantially different from those reported.

## 4. Discussion

This systematic review and meta-analysis analysis demonstrated that the addition of existing exercise interventions in children did not result in significant improvements to bone health. This review was only able to identify a single study that assessed the effect on bone health in pre-school children (aged between 2–5 years) and they did not report sufficient data for analysis. Finally, due to risk of bias due to deviations from the intended interventions and measurement of outcomes, all studies were at high risk of bias and the overall quality of the evidence was very low.

Femoral neck BMC significantly improved at <15-month follow-up with additional exercise interventions in children, however this result did not remain significant following sensitivity analysis. Whole-body and lumbar spine BMC did not significantly change at any follow-up timepoint with the additional exercise interventions. Additional exercise interventions also failed to significantly improve aBMD at the femoral neck, lumbar spine or whole-body.

No significant improvements were observed in tibial vBMD at any level with additional exercise interventions. However, vBMD may not be an overly specific measure and has been shown to be unable to differentiate between female and male Australian Football players [56]. Despite significant differences between Australian Football Men’s and Women’s players according to other musculoskeletal morphological characteristics (e.g., bone mass, cross-sectional area and robustness), vBMD (measured via pQCT) was unable to differentiate between cohorts. Furthermore, despite being one of the universal measures of hard-tissue health, BMD may have limited utility in evaluating bone health and strength [57].

A number of additional measures of bone strength were assessed using pQCT. No significant improvements were observed in trabecular vBMD, CSA, cortical area, cortical thickness or SSIPOL with additional exercise interventions. This was a relatively unexpected finding as longitudinal exercise has resulted in favourable hard-tissue improvements in adult athletes previously [56,58]. Specifically, elite male athletes exhibit superior hard-tissue characteristics in their support leg versus their kicking leg, which is likely due to the different loading patterns between limbs [56]. The support limb typically experiences more frequent impacts as it acts as the stabilising leg during kicking actions and is typically the dominant single-leg jumping and landing leg [58]. Furthermore, greater hard-tissue asymmetry between kicking and support limbs was found in experienced athletes over younger inexperienced athletes, further illustrating the positive effect of longitudinal specific exercise loading on bone health. However, the addition of exercise interventions in children within this systematic review did not result in favourable improvements in hard-tissue characteristics.

These findings suggest, at least in children aged 6–12 years, that either normal growth and development, or standard exercise associated with school and childhood may be sufficient to increase the hard-tissue characteristics of bone, as significant within-group improvements over time were observed in both intervention and control groups in all studies. It may be that the magnitude of change in hard-tissue characteristics through standard exercise and normal human physical development was adequate and any additional exercise interventions could not produce any further improvements in hard-tissue as the physiological plateau was already reached. Alternatively, another explanation is that the additional exercise interventions were insufficient in producing positive osteogenic effects beyond what children were already being exposed to in standard exercise programs. However, the lack of data in children aged 2–5 years was unexpected as this is a group that have not yet commenced formal exercise interventions within school.

The results of our review do not align with the conclusions of previous systematic reviews in this field, with sharp contrasts in the findings of our meta-analyses. One reason might be that the meta-analysis performed by Behringer et al. 2014 [3] pooled all studies that measured bone health (e.g., pooled different anatomical locations), with follow-up ranging from 6–208 weeks, creating a much larger sample (2985 participants including 1640 intervention participants) providing more statistical power to analysis, whereas our largest meta-analysis contained 915 total participants. However, we feel that breaking up timepoints and regions as we have in this review, provides a far more accurate representation of the outcomes from exercise interventions.

No difference in fracture incidence was observed with additional exercise interventions in children in the single trial that recorded fracture incidence. This may be due to the lack of observable increases in bone health observed within our meta-analysis. Alternatively, other measures of bone strength not measured by either DXA or pQCT may have a greater link with fracture incidence. The use of nuclear magnetic resonance spectroscopy, which provides a far more detailed overview of bone structure and quality when compared to standard DXA or pQCT, might be informative in future studies. Alternatively, high-resolution pQCT (HR-pQCT) measures of hard-tissue characteristics were found to predict fracture in two separate systematic reviews [59,60]. However, prediction of fracture was observed to be stronger in the radius, rather than the tibia [59]. This may be problematic as radius fracture is likely the result of contact/impact, rather than overuse, which is distinct from the tibia, being a weight-bearing bone. It must also be noted that the lack of fracture incidence in children undertaking standard exercise may be due to the increased exercise levels being inherently associated with increased exposure to potentially risky physical activities (e.g., basketball), placing the children undertaking additional exercise interventions at higher risk of fracture.

With most studies at high risk of bias due to deviations from the intended interventions, future research should attempt to design and report their interventions in accordance with the TIDieR [39] and/or CERT checklists [48]. This will increase transparency [61,62] and ensure any tailoring or modifications to interventions are planned, recorded and accounted for within any analysis. Furthermore, adherence, including adherence to the control group protocol, was not recorded in all studies or factored into analysis, and this may have influenced outcomes [61,62]. In addition to adherence to the planned intervention, general physical activity should be considered to influence the results and future studies using activity trackers might provide greater insight into the role of exercise interventions for bone health.

Adverse events are an essential reporting element of clinical trials [63] but were not reported within included studies. Subsequently, not allowing us to discuss any potential adverse events associated with additional exercise interventions to inform discussion on the benefits of its implementation. One of the main drivers of apophysitis or bone stress injury is an overload of impact exercise [64]. Therefore, studies that increase exercise levels beyond standard physical activity should report adverse events. For example, if a future study was to demonstrate a small effect for additional exercise interventions in bone health, the benefits of implementing this study need to balance how many participants presented with injuries, such as apophysitis, as a direct result of the intervention to determine if it is worthwhile.

### Limitations

Several studies did not respond to requests for missing data or advised that data was no longer available, which was expected due to the age of many included studies, with measures of variability being inputted when data was missing as per Cochrane recommendations [38].

Unfortunately, not all studies provided separate outcomes by participant sex, which is important due to bone growth rate differences. However, given all but one study was randomized (and the non-randomized study provided separate data for boys and girls) we would not expect this to have influenced the results.

The DXA and pQCT procedures were not consistently reported between studies, and some differences did exist in assessment methodologies. However, given our meta-analysis included between-group differences (with the procedure for all groups within an individual study being the same), we would not expect this to have influenced the results.

## 5. Conclusions

The addition of exercise interventions, beyond what is provided to children, does not appear to improve DXA and pQCT measures of bone health. The effect of additional exercise interventions on bone health in pre-school children is largely unknown. Future trials should ensure adherence is clearly reported and controlled for within analysis as well as including reports of adverse events (e.g., apophysitis) that occurs due to increased exercise interventions.

## Figures and Tables

**Table 1:** Summary of included studies.

Study	Location	Design	Population	Sample Size (*n*)	Ages	Intervention Type	Group	Age, Mean (SD) Years	Height, Mean (SD) cm	Weight, Mean (SD) kg
Alwis. 2008	Sweden	Cluster RCT	Healthy	103	Children	Specific impact loading	INT	7.8 (0.6)	129.4 (6.5)	28.4 (5.8)
						Standard physical activity	CON	8.0 (0.6)	130.1 (6.7)	27.6 (5.4)
Anliker. 2012	Switzerland	Cluster RCT	Healthy	45	Children	Specific impact loading	INT	10.5 (1.2)	140 (12.0)	34.6 (7.7)
						Standard physical activity	CON	10.8 (1.1)	143 (7.0)	34 (5.7)
Barbeau. 2007	United States of America	RCT	Healthy	201	Children	General Physical Activity	INT	9.5 (NR)	NR	NR
						Standard physical activity	CON	9.5 (NR)	NR	NR
Daly. 2016	Australia	Cluster RCT	Healthy	727	Children	General Physical Activity	INT (Girls)	8.1 (0.3)	128.4 (5.5)	28.6 (5.9)
						General Physical Activity	INT (Boys)	8.1 (0.4)	130.4 (5.5)	28.9 (5.2)
						Standard physical activity	CON (Girls)	8.1 (0.4)	129 (5.3)	28.9 (5.7)
						Standard physical activity	CON (Boys)	8.2 (0.3)	129.9 (5.8)	28.7 (5.3)
Fuchs. 2001	United States of America	RCT	Healthy	89	Children	Specific impact loading	INT	7.5 (0.2)	125.1 (1.3)	27.1 (0.8)
						Standard physical activity	CON	7.6 (0.2)	126.8 (1.2)	28.0 (1.0)
Gutin. 2008	United States of America	Cluster RCT	Healthy	617	Children	General Physical Activity	INT	NR	NR	NR
						Standard physical activity	CON	NR	NR	NR
Hasselstrom. 2008	Denmark	Non-RCT	Healthy	704	Children	General Physical Activity	INT (Girls)	6.7 (0.3)	121.5 (4.9)	23.6 (3.2)
						General Physical Activity	INT (Boys)	6.8 (0.4)	124.2 (4.5)	24.4 (3.0)
						Standard physical activity	CON (Girls)	6.7 (0.4)	122.5 (4.6)	23.9 (3.8)
						Standard physical activity	CON (Boys)	6.8 (0.4)	123.6 (5.2)	24.7 (3.6)
Larsen. 2016	Denmark	Cluster RCT	Healthy	295	Children	Team Impact Sport	INT A (Girls)	9.3 (0.3)	137.6 (7.1)	33.0 (8.2)
						Team Impact Sport	INT A (Boys)	9.3 (0.4)	139.2 (6.4)	32.6 (5.4)
						Specific impact loading	INT B (Girls)	9.3 (0.3)	136 (5.7)	32.2 (6.7)
						Specific impact loading	INT B (Boys)	9.2 (0.4)	138.7 (5.5)	32.2 (7.3)
						Standard physical activity	CON (Girls)	9.4 (0.3)	138.7 (6.5)	33.5 (6.7)
						Standard physical activity	CON (Boys)	9.3 (0.3)	137.9 (5.3)	31.7 (5.2)
Macdonald. 2007	Canada	Cluster RCT	Healthy	410	Children	Specific impact loading	INT (Girls)	10.2 (0.6)	141.5 (7.5)	36.3 (8.4)
						Specific impact loading	INT (Boys)	10.2 (0.6)	141.5 (7.2)	37.2 (9.3)
						Standard physical activity	CON (Girls)	10.3 (0.5)	140.2 (7.5)	35.2 (8.7)
						Standard physical activity	CON (Boys)	10.3 (0.6)	141.2 (6.8)	39.7 (9.6)
MacKelvie. 2001	Canada	Cluster RCT	Healthy	198	Children	Specific impact loading	INT (Pre-pubertal Girls)	10.0 (0.6)	138.6 (7.6)	31.2 (6.1)
						Specific impact loading	INT (Early-pubertal Girls)	10.4 (0.7)	143.8 (7.7)	39.1 (8.3)
						Specific impact loading	INT (Boys)	10.2 (0.6)	140.6 (6.0)	35.5 (8.3)
						Standard physical activity	CON (Pre-pubertal Girls)	10.1 (0.5)	137.3 (6.2)	31.1 (5.6)
						Standard physical activity	CON (Early-pubertal Girls)	10.5 (0.6)	145.6 (6.4)	41.3 (8.3)
						Standard physical activity	CON (Boys)	10.3 (0.7)	141.8 (7.1)	36.6 (10.1)
McKay. 2000	Canada	Cluster RCT	Healthy	144	Children	Specific impact loading	INT	NR	133.9 (0.7)	30.5 (0.8)
						Standard physical activity	CON	NR	135.1 (1.1)	30.8 (1.0)
Meiring. 2014	South Africa	Cluster RCT	Healthy	22	Children	Standard physical activity	CON	9.3 (0.9)	135.1 (8.2)	30.6 (4.7)
						Specific impact loading	INT	9.7 (1.2)	135.9 (8.7)	30.0 (5.1)
Meyer. 2011	Switzerland	Cluster RCT	Healthy	291	Children	Specific impact loading	INT	8.8 (2.1)	133.3 (13.1)	30.7 (8.7)
						Standard physical activity	CON	8.8 (2.2)	134.2 (14.2)	30.4 (9.8)
Nogueira. 2014	Australia	Cluster RCT	Healthy	138	Children	Specific impact loading	INT	10.5 (0.6)	144.2 (6.7)	39.3 (9.4)
						Standard physical activity	CON	10.7 (0.6)	142.5 (7.1)	37.2 (7.2)
Specker. 2004	United States of America	RCT	Healthy	161	Preschool Children	Specific impact loading	INT	3.8 (0.5)	100.6 (6.1)	16.3 (2.2)
						Standard physical activity	CON	4.0 (0.6)	102.4 (5.4)	16.9 (2.3)
Staiano. 2018	United States of America	RCT	Overweight/Obese	46	Children	Specific impact loading	INT	NR	NR	NR
						Standard physical activity	CON	NR	NR	NR

Legend: RCT = randomised controlled trial, Children = 6–12 years, Preschool Children = 2–5 years, MVPA = Moderate to vigorous physical activity, POP = Pediatric Osteoporosis Prevention, INT = Intervention. CON = Control, NR = not reported.

**Figure A1:**
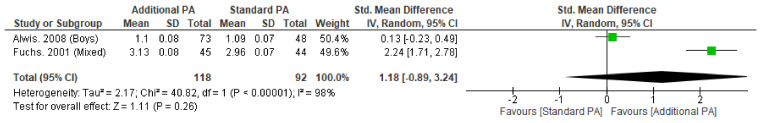
Effect of Additional Exercise Interventions on Femoral Neck Cross-Sectional Area at Less than 15 Months Follow-up. Legend: PA = Physical Activity, Std = standardised, IV = inverse variance, SD- standard deviation, CI = confidence interval.

**Figure A2:**
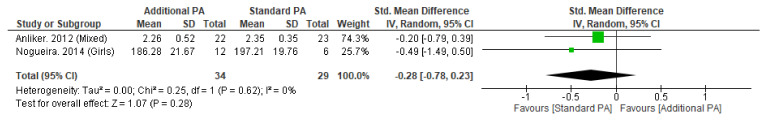
Effect of Additional Exercise Intervention on Volumetric Bone Mineral Content within the Tibia (4%) at Less than 12 Months Follow-up. Legend: PA = Physical Activity, Std = standardised, IV = inverse variance, SD- standard deviation, CI = confidence interval.

**Figure A3:**
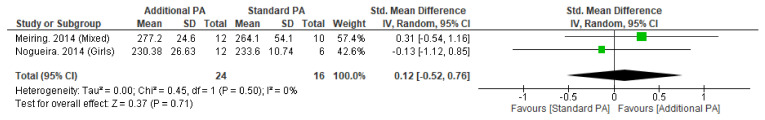
Effect of additional exercise interventions on trabecular bone mineral density within the Tibia (4%) at less than 12 months follow-up. Legend: PA = Physical Activity, Std = standardised, IV = inverse variance, SD- standard deviation, CI = confidence interval.

**Figure A4:**
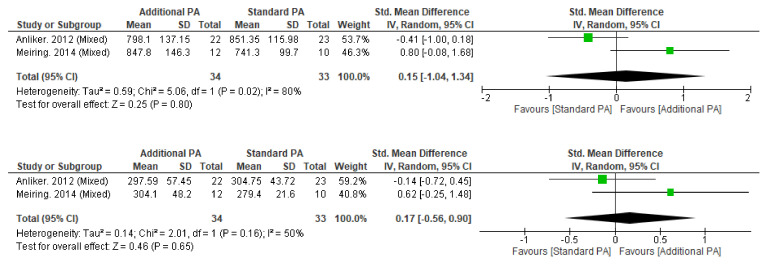
Effect of Additional Exercise Interventions on bone cross-sectional area at less than 12 months follow-up measures. (**Top**) 4% Tibia, (**Bottom**) 38%Tibia. Legend: PA = Physical Activity, Std = standardised, IV = inverse variance, SD- standard deviation, CI = confidence interval.

**Figure A5:**
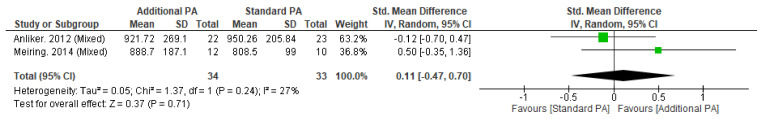
Effect of additional exercise interventions on the polar stress–strain index within the Tibia (38%) at less than 12 months follow-up. Legend: PA = Physical Activity, Std = standardised, IV = inverse variance, SD- standard deviation, CI = confidence interval.

## Data Availability

The complete dataset for this review is presented across the tables and appendices.

## Appendix A. Systematic Review Search Strategy

**Search Item**	**Combiners**	**Terms**
1	Problem of Interest	Fracture OR dual-energy x-ray absorptiometry OR DXA OR bone mineral density OR peripheral Quantitative Computed Tomography OR pQCT OR bone density OR trabecular area OR cortical area OR bone mineral content OR periosteal size OR endosteal size OR cortical thickness OR bone mass OR bone strength OR skeletal age assessment
2	Participants	Child* OR youth* OR juvenile OR minor OR kid
3	Participants	Physical OR activity OR exercise OR athletics OR recreation OR play OR games OR training OR gym OR resistance training OR workout OR practice
4	Exclusion	review OR meta-analysis
5		Number #1 AND #2 AND #3 NOT #4
	Limitations	Clinical Trial, Humans, English, Child, Preschool Child

## Appendix B. Search Strategy Documentation

**Database**	**Date of Search**	**Search Strategy Used (Keywords & Boolean)**	**Search Limits or Filters (e.g., Dates, Language)**	**Number of Results Found**	**Comments**
PUBMED	3 May 2022	(Child * OR youth * OR juvenile OR minor OR kid) AND (Physical OR activity OR exercise OR athletics OR recreation OR play OR games OR training OR gym OR resistance training OR workout OR practice) AND (Fracture OR dual-energy X-ray absorptiometry OR DXA OR bone mineral density OR peripheral Quantitative Computed Tomography OR pQCT OR bone density OR trabecular area OR cortical area OR bone mineral content OR periosteal size OR endosteal size OR cortical thickness OR bone mass OR bone strength OR skeletal age assessment) NOT (review OR meta-analysis)	Clinical Trial, Humans, English, Child (6–12), Preschool Child (2–5), Title/abstract	236	Exported to End Note
CINAHL (Full Text)	3 May 2022	(Child * OR youth * OR juvenile OR minor OR kid) AND (Physical OR activity OR exercise OR athletics OR recreation OR play OR games OR training OR gym OR resistance training OR workout OR practice) AND (Fracture OR dual-energy X-ray absorptiometry OR DXA OR bone mineral density OR peripheral Quantitative Computed Tomography OR pQCT OR bone density OR trabecular area OR cortical area OR bone mineral content OR periosteal size OR endosteal size OR cortical thickness OR bone mass OR bone strength OR skeletal age assessment) NOT (review OR meta-analysis)	Research article, Humans, Peer-reviewed, English, Child (6–12), Preschool Child (2–5), Title	53	Exported to End Note
CENTRAL	3 May 2022	(Child * OR youth * OR juvenile OR minor OR kid) AND (Physical OR activity OR exercise OR athletics OR recreation OR play OR games OR training OR gym OR resistance training OR workout OR practice) AND (Fracture OR dual-energy X-ray absorptiometry OR DXA OR bone mineral density OR peripheral Quantitative Computed Tomography OR pQCT OR bone density OR trabecular area OR cortical area OR bone mineral content OR periosteal size OR endosteal size OR cortical thickness OR bone mass OR bone strength OR skeletal age assessment) NOT (review OR meta-analysis)	Trial, title	55	Exported to End Note
SportsDISCUS	3 May 2022	(Child * OR youth * OR juvenile OR minor OR kid) AND (Physical OR activity OR exercise OR athletics OR recreation OR play OR games OR training OR gym OR resistance training OR workout OR practice) AND (Fracture OR dual-energy X-ray absorptiometry OR DXA OR bone mineral density OR peripheral Quantitative Computed Tomography OR pQCT OR bone density OR trabecular area OR cortical area OR bone mineral content OR periosteal size OR endosteal size OR cortical thickness OR bone mass OR bone strength OR skeletal age assessment) NOT (review OR meta-analysis)	Peer-reviewed, title	40	Exported to End Note
Web of Science	3 May 2022	(((TI = (Fracture OR dual-energy X-ray absorptiometry OR DXA OR bone mineral density OR peripheral Quantitative Computed Tomography OR pQCT OR bone density OR trabecular area OR cortical area OR bone mineral content OR periosteal size OR endosteal size OR cortical thickness OR bone mass OR bone strength OR skeletal age assessment)) AND TI = (Child * OR youth * OR juvenile OR minor OR kid)) AND TI = (Physical OR activity OR exercise OR athletics OR recreation OR play OR games OR training OR gym OR resistance training OR workout OR practice)) NOT TI = (review OR meta-analysis)	Nil	243	Exported to End Note
Key Journals	3 May 2022	N/A	N/A	1	
TOTAL					628

## Appendix C. Study-by-Study Exclusion Judgements

**Study Author**	**Year**	**Title**	**Exclusion Reason**
Allerton	2008	Physical Activity is Positively Related to Bone Mineral Density in Prepubertal Youth.	Conference abstract
Clark	2007	Vigorous physical activity at age 9 increases the risk of childhood fractures, despite increasing bone mass.	Conference abstract
Cole	2010	Physical activity is associated with increased volumetric bone density and bone strength in early childhood.	Conference abstract
Coster	2014	Increased Physical Activity during Growth Improves Muscular Development without Affecting Fracture Risk—a Four-Year Prospective Controlled Exercise Intervention Study in 2525 Children.	Conference abstract
Fritz	2014	A School-based Seven Year Exercise Intervention Program in 6–9-Year-Old Children Improve Skeletal Traits without Increasing the Fracture Risk—A Population-Based Prospective Controlled Study in 3534 Children.	Conference abstract
Fritz	2015	Increased physical activity in childhood reduces fracture risk—An 8-year intervention study in 3534 children.	Conference abstract
Janz	2003	Physical activity intensities and femoral neck strength in young children: The Iowa bone development study.	Conference abstract
MacKelvie	2003	A school-based exercise intervention elicits substantial bone health benefits: a 2-year randomized controlled trial in girls.	Conference abstract
Naka	2005	A two-year longitudinal study on the relationship between sports activity and bone mass gain in Japanese boys and girls: Kyoto Kids Increase Density in the Skeleton Study (Kyoto KIDS Study).	Conference abstract
Naka	2007	A two-year longitudinal study on the relationship between the effective time of sports activity and bone mineral density in Japanese girls: Kyoto kids increase density in the skeleton study (Kyoto KIDS Study).	Conference abstract
Tamaki	2005	Effect of physical activity in elementary school on bone mineral density in Japanese children and adolescents: A 3-year longitudinal study.	Conference abstract
Tamaki	2006	Which element of physical activity is more important for determining peak bone mass in Japanese children and adolescents, the period, frequency, or active duration per each activity?	Conference abstract
Cohen	2017	Bone Health is Maintained, While Fat Mass is Reduced in Pre-pubertal Children with Obesity Participating in a 1-Year Family-Centered Lifestyle Intervention.	Wrong intervention (included dietary supplementation)
Yu	2005	Effects of strength training on body composition and bone mineral content in children who are obese.	Wrong intervention (included dietary supplementation)
Alberga	2013	The effects of resistance exercise training on body composition and strength in obese prepubertal children.	Wrong outcomes (did not include bone outcomes)
Comeras-Chueca	2022	Active Video Games Improve Muscular Fitness and Motor Skills in Children with Overweight or Obesity.	Wrong outcomes (did not include bone outcomes)
Davis	2012	Exercise dose and diabetes risk in overweight and obese children: a randomized controlled trial.	Wrong outcomes (did not include bone outcomes)
Dias	2018	Effect of High-Intensity Interval Training on Fitness, Fat Mass and Cardiometabolic Biomarkers in Children with Obesity: A Randomised Controlled Trial.	Wrong outcomes (did not include bone outcomes)
Klakk	2013	Effect of four additional physical education lessons on body composition in children aged 8–13 year—a prospective study during two school years.	Wrong outcomes (did not include bone outcomes)
Yin	2012	The impact of a 3-year after-school obesity prevention program in elementary school children.	Wrong outcomes (did not include bone outcomes)

## Appendix D. Study funding and conflicts of interest

**Study**	**Funding**	**Conflict of Interest**
Alwis. 2008	Swedish Research Council, Centre for Athletic Research, Osterlund Foundation, Kock Foundation, Region Skane Foundation.	None declared.
Anliker. 2012	Swiss Federal Sports Commission Grant.	None declared.
Barbeau. 2007	National Institutes of Health (NIH) Grant.	None declared.
Daly. 2016	Commonwealth Education Trust, Canberra Hospital Salaried Staff Specialists.	None declared.
Fuchs. 2001	National Institute of Health Grant, Division of National Institute of Arthiritis and Musculoskeletal diseases.	None declared.
Gutin. 2008	National Institute of Health (NIH).	None declared.
Hasselstrom. 2008	Danish Heart Foundation, National Board of Health, Danish Ministry of Health, Danish Ministry of Culture, Danish Sport Association.	None declared.
Larsen. 2016	Nordea Fonden, Aase and Ejnar Danielsens Foundation, Augustinus Fonden, FIFA Medical Assessment and Research Centre, Danish Football Association, Danish Ministry of Culture.	None declared.
Macdonald. 2007	British Columbia Ministry of Health, 2010 Legacies Now, BC Ministry of Tourism, Sport and Arts, Canadian Institutes of Health Research.	None declared.
MacKelvie. 2001	British Columbia Health Research Foundation, Michael Smith Foundation for Health Research.	None declared.
McKay. 2000	Canadian Institutes of Health Research and the Michael Smith Foundation for Health Research.	None declared.
Meiring. 2014	Carnegie Corporation, Medical Research Council of South Africa, Sugar Association of South Africa.	None declared.
Meyer. 2011	Swiss Federal Office of Sports, Swiss National Science Foundation.	None declared.
Nogueira. 2014	None declared.	None declared.
Specker. 2004	National Institutes of Health Grant.	None declared.
Staiano. 2018	American Heart Association, NORC Centre, National Institute of Diabetes and Digestive and Kidney Diseases.	None declared.

## Appendix E. Template for Intervention Description and Replication (TIDieR) Checklist Items

**Study**	**Materials**	**Procedures**	**Provider**	**Delivery**	**Intervention location**	**Dosage (Sets, Reps)**	**Dosage (Frequency/Week)**	**Dosage (Duration in Weeks)**	**Tailoring**	**Modifications**	**Adherence**
Alwis. 2008	None	Extra Activity, running, jumping, climbing to 200min/week	School teachers	Face to face	Unspecified	NR	5/week	2 years	No	No	N/A
Anliker. 2012	None	10 min jumping	School teachers	Face to face—class setting	Unspecified	60–150 jumps	2/week	36	No	No	N/A
Barbeau. 2007	None	Skills development, MVPA, stretching, toning	Classroom teachers, assistant teachers and a research staff member	Face to face—class setting	Unspecified	NR	5/week	40 weeks	No	No	Students could not miss 3 sessions in a row
Daly. 2016	None	General PA	Specialist Teacher from Bluearth Foundation	Face to face—class setting	Not specified	NR	2/week	35 weeks/year for 4 years	No	No	High
Fuchs. 2001	61cm Box	Current practice plus 20 min—3 times/week	Laboratory Researcher + 4 trained instructors	Face to Face	Gym	100 jumps per session	3 times/week for 20 min	7 months	No	50 jumps per session were started with, and slowly progressed to 100 jumps by the 5th week	N/A
Gutin. 2008	None	80 min/day, 5 days per week	Qualified Physical Trainers	Face-to-face	Gym	NR	5/week	3 years	No	No	44%
Hasselstrom. 2008	None	Extra PE classes—180 min/week	Qualified Physical Trainers	Face-to-face	School	NR	2/week	3 years	No	No	N/A
Larsen. 2016 (Small-sided games)	None	3 v 3 football, basketball, floorball or other	Qualified University Trainers	Face-to-face	School	NR	3/week	10 months	No	No	N/A
Larsen. 2016 (Circuit training)	None	50% was 30 s effort, 45 s rest, 6–10 stations. Plyos, strength, etc. 50% was games with strength, etc.	Qualified University Trainers	Face-to-face	School	NR	3/week	10 months	No	No	N/A
Macdonald. 2007	Classroom Action Bin	Standard curriculum—2 × 40 min PE classes + 15 min per day of random activities + 9 min per day of jumping activities	Normal teachers	Face-to-face	School	NR	5/week (school days)	11 months dosage (14 months until follow-up)	No	No	No
MacKelvie. 2001	None	School curriculum with 10–12 min diverse weight bearing activity during normal class, plus 10–12 min on an extra day. Activities included circuit training with 5 different jumping activities with GRF that ranged from 3.5–5 times body weight. Children progressed from 50 to 100 jumps per session across 10 weeks. A higher proportion of high-impact jumps were utilised in the second year of intervention	Normal teachers	Face to face	School	50–100 jumps per session	3/week	20 months	No	No	No
McKay. 2000	None	School curriculum with 10–30 min within each PE class + one extra day—minimum 10 min loading which focused on jumping. 10 DBL tuck jumps before each class. Bench jumping and circuit training were added as the intervention progressed	Classroom teachers	Face to face	School	10 tuck jumps to start the class + exercise intervention	3/week	8 months	No	No	N/A
Meiring. 2014	None	School curriculum + 2 × 45 min sessions—Exercise circuit with 5 activities	Classroom teachers or after school care teachers	Face-to-face	Sports field	45 min	2/week	20 weeks	No	No	N/A
Meyer. 2011	None	School curriculum + 2 additional PE classes (5 total)—At least 10 min of jumping activities. Additionally, included short activity breaks in the middle of normal class, plus students had physical activity homework to complete	School teachers	Face to face	School + home	NR	5/week	9 months	No	No	N/A
Nogueira. 2014	None	School curriculum + 10 min of continuous high-intensity movements of medium to high impact at various speeds—jumps, hops, squats, lunges, handstands, cartwheels	Trained Instructor	Face to face	School	NR	3/week	9 months	No	No	N/A
Specker. 2004	None	30 min of moderate vigorous activity, which focused on jumping, hopping, skipping on every school day	Childcare teachers	Face to face	Childcare/School	NR	5/week	12 months	No	No	72%
Staiano. 2018	Kinect and XBOX 360 gaming console, 24-week XBOX live subscription and 4 exercise games	3 times weekly playing exergames on XBOX—10 min per session in Week 1, increasing by 10 min each session each week, sustaining at 60 min per session after Week 6	Parent/Guardian	Face to face	Home	NR	3/week	24 weeks	No	No	94.40%

## Appendix F. Dual X-ray Absorptiometry

**Outcome**	**Timepoint (Weeks)**	**Study**	**Girls (Baseline)- Mean (SD), *n***		**Girls (Follow-up)- Mean (SD), *n***		**Boys (Baseline)- Mean (SD), *n***		**Boys (Follow-up)- Mean (SD), *n***		**All (Baseline)- Mean (SD), *n***		**All (Follow-up)- Mean (SD), *n***	
			**INT**	**CON**	**INT**	**CON**	**INT**	**CON**	**INT**	**CON**	**INT**	**CON**	**INT**	**CON**
BMC (Whole Body)	20	Meiring. 2014									778.40 (164.00), 12	753.70 (103.60), 10	822.60 (195.50), 12	792.90 (116.70), 10
BMC (Whole Body)	32	McKay. 2005									1054.00 (97.00), 51	1047.00 (169.00), 73	1146.70 (NR), 51	1153.00 (NR), 73
BMC (Whole Body)	36	Nogueira. 2014	1523.67 (225.67), 12	1561.67 (87.44), 6	1732.80 (314.97), 12	1701.50 (117.21), 6								
BMC (Whole Body)	40	Barbeau. 2007	1260.00 (310.00), 118	1230.00 (280.00), 83	1460.00 (360.00), 118	1400.00 (330.00), 83								
BMC (Whole Body)	52	Alwis. 2008					991.00 (84.00), 80	980.00 (74.00), 57	1135.00 (NR), 78	1127.00 (NR), 54				
BMC (Whole Body)	60	Macdonald. 2008	1040.30 (213.20), 142	1003.50 (159.10), 55	1253.20 (263.60), 142	1216.30 (213.80), 55	1078.60 (193.10), 151	1098.10 (178.10), 62	1260.30 (237.60), 151	1259.90 (206.60), 62				
BMC (Whole Body)	104	Linden. 2006	939.00 (153.00), 49	933.00 (181.00), 50	1245.00 (240.00), 49	1204.00 (245.00), 50								
BMC (Whole Body)	156	Meyer. 2013									732.00 (223.00), 149	750 (257.00), 65	1196.00 (458.00), 149	1153.00 (424.00), 65
BMC (Whole Body)	208	Daly. 2016	886.00 (101.00), 192	909.00 (114.00), 170	1362.00 (NR), 94	1364.00 (NR), 69	933.00 (116.00), 206	956.00 (117.00), 159	1342.00 (NR), 97	1365.00 (NR), 76				
BMC (Whole Body)	364	Fritz. 2016b	614.00 (132.00), 72	607.00 (142.00), 45	1828.00 (NR), 72	1745.00 (NR), 45	646.00 (151.00), 100	671.00 (144.00), 47	1986.00 (NR), 100	2005.00 (NR), 47				
BMC (Whole Body)	416	Coster. 2016	609.00 (132.00)	612.00 (145.00), 39	1986.00 (365.00), 65	1840.00 (384.00), 39	650.00 (151.00), 93	681.00 (140.00), 37	2210.00 (513.00), 93	2292.00 (473.00), 37				
BMC (Total Hip)	20	Meiring. 2014									17.60 (4.90), 12	16.30 (2.90), 10	18.70 (5.50), 12	16.50 (3.10), 10
BMC (Total Hip)	156	Meyer. 2013									14.37 (5.59), 149	14.72 (5.69), 65	24.98 (10.80), 149	23.53 (8.37), 65
BMC (Total Hip)	364	Gunter. 2008	10.60 (2.96), 14	10.10 (1.37), 8	31.26 (5.4), 11	30.15 (3.38), 7	11.64 (2.33), 19	10.89 (2.53), 16	39.93 (9.56), 18	39.31 (9.93), 13				
BMC (Radius)	20	Meiring. 2014									3.60 (0.80), 12	3.40 (0.50), 10	3.80 (0.80), 12	3.60 (0.60), 10
BMC (Ulna)	20	Meiring. 2014									2.50 (0.60), 12	2.30 (0.40), 10	2.70 (0.60), 12	2.50 (0.40), 10
BMC (Forearm)	156	Hasselstrom. 2008	1.75 (0.26), 135	1.77 (0.29), 76	2.14 (0.34), 135	2.12 (0.40), 76	1.92 (0.30), 135	1.97 (0.31), 62	2.33 (0.38), 135	2.35 (0.45), 62				
BMC (Calcaneus)	156	Hasselstrom. 2008	0.77 (0.23), 135	0.77 (0.21), 76	1.26 (0.27), 135	1.29 (0.25), 76	0.75 (0.24), 135	0.8 (0.23), 62	1.38 (0.25), 135	1.35 (0.26), 62				
BMC (Leg)	104	Linden. 2006	283.90 (60.70), 49	282.70 (72.50), 50	430.30 (105.10), 49	409.80 (106.60), 50								
BMC (Inter- trochanteric)	32	McKay. 2005									10.60 (2.20), 51	10.20 (2.20), 73	12.10 (NR), 51	11.40 (NR), 73
BMC (Greater trochanter)	32	McKay. 2005									2.90 (0.90), 51	2.70 (0.80), 73	3.50 (NR), 51	3.25 (NR), 73
BMC (Greater trochanter)	364	Gunter. 2008	2.47 (0.84), 14	2.13 (0.54), 8	8.31 (1.42), 11	7.49 (1.05), 7	2.54 (0.68), 19	2.24 (0.59), 16	11.11 (2.62), 18	10.91 (2.54), 13				
BMC (Proximal Femur)	32	McKay. 2005									16.20 (3.40), 51	15.60 (3.20), 73	18.50 (NR), 51	17.50 (NR), 73
BMC (Proximal Femur)	60	Macdonald. 2008	15.20 (3.50), 142	14.40 (2.90), 55	19.00 (4.50), 142	18.40 (4.00), 55	16.10 (3.40), 151	16.00 (3.00), 62	19.60 (4.50), 151	19.60 (3.90), 62				
BMC (Femoral Neck)	20	Meiring. 2014									2.90 (0.50), 12	2.70 (0.40), 10	3.00 (0.50), 12	2.70 (0.3), 10
BMC (Femoral Neck)	28	Fuchs. 2001									1.84 (0.07), 45	1.82 (0.06), 44	2.00 (0.07), 45	1.89 (0.06), 44
BMC (Femoral Neck)	32	McKay. 2005									2.70 (0.40), 51	2.60 (0.40), 73	2.89 (NR), 51	2.80 (NR), 73
BMC (Femoral Neck)	36	Nogueira. 2014	2.77 (0.45), 12	3.05 (0.28), 6	3.11 (0.56), 12	3.24 (0.39), 6								
BMC (Femoral Neck)	52	Alwis. 2008					2.90 (0.60), 80	2.8 (0.50), 57	3.13 (NR), 76	3.08 (NR), 51				
BMC (Femoral Neck)	60	Macdonald. 2008	2.52 (0.42), 142	2.47 (0.38), 55	2.89 (0.52), 142	2.80 (0.50), 55	2.79 (0.43), 151	2.83 (0.41), 62	3.08 (0.48), 151	3.10 (0.48), 62				
BMC (Femoral Neck)	104	Linden. 2006	2.60 (0.60), 49	2.70 (0.60), 50	3.30 (0.60), 49	3.20 (0.60), 50								
BMC (Femoral Neck)	156	Meyer. 2013									2.33 (0.77), 149	2.39 (0.78), 65	3.54 (0.92), 65	3.67 (1.03), 149
BMC (Femoral Neck)	364	Gunter. 2008	1.59 (0.37), 14	1.50 (0.37), 8	4.18 (0.53), 11	4.21 (0.53), 7	1.89 (0.38), 19	1.79 (0.40), 16	4.92 (0.88), 18	4.72 (0.93), 13				
BMC (Femoral Neck)	364	Fritz. 2016b	2.50 (0.50), 72	2.60 (0.60), 45	4.90 (NR), 72	4.60 (NR), 45	2.80 (0.60), 100	2.80 (0.50), 47	5.10 (NR), 100	5.20 (NR), 47				
BMC (Femoral Neck)	364	Fritz. 2016c									2.70 (0.60), 172	2.70 (0.60), 92	5.10 (NR), 172	4.90 (NR), 92
BMC (Femoral Neck)	416	Coster. 2016	2.50 (0.60), 65	2.60 (0.60), 39	5.20 (0.90), 65	4.80 (1.10), 39	2.80 (0.60), 93	2.90 (0.50), 37	5.60 (1.10), 93	5.80 (1.00), 37				
BMC (L3)	52	Alwis. 2008					5.30 (1.10), 80	5.60 (1.00), 57	6.02 (NR), 76	6.18 (NR), 51				
BMC (L3)	104	Linden. 2006	5.00 (1.00), 49	5.30 (1.20), 50	6.70 (1.60), 49	6.30 (1.40), 50								
BMC (Lumbar Spine)	20	Meiring. 2014									23.10 (4.60), 12	23.40 (4.60), 10	24.30 (6.20), 12	24.40 (4.70), 10
BMC (Lumbar Spine)	28	Fuchs. 2001									20.10 (0.51), 45	20.39 (0.55), 44	22.06 (0.57), 45	21.64 (0.58), 44
BMC (Lumbar Spine)	32	McKay. 2005									24.60 (5.10), 51	24.30 (4.40), 73	27.20 (NR), 51	27.10 (NR), 73
BMC (Lumbar Spine)	36	Nogueira. 2014	20.40 (4.20), 12	21.10 (3.00), 6	24.80 (7.20), 12	23.20 (3.60), 6								
BMC (Lumbar Spine)	60	Macdonald. 2008	24.00 (6.10), 142	23.20 (4.90), 55	30.80 (8.40), 142	29.6 (7.00), 55	23.10 (4.90), 151	23.60 (4.30), 62	27.30 (6.40), 151	27.20 (5.10), 62				
BMC (Lumbar Spine)	104	Linden. 2006	15.20 (3.10), 49	15.50 (3.30), 50	19.70 (4.90), 49	19.00 (4.00), 50								
BMC (Femoral Neck)	156	Meyer. 2013									22.26 (6.10), 149	22.99 (7.09), 65	39.07 (16.09), 149	37.86 (14.99), 65
BMC (Lumbar Spine)	364	Fritz. 2016b	82.40 (19.10), 72	77.00 (17.60), 45	242.6 (NR), 72	227.80 (NR), 45	84.80 (21.40), 100	85.20 (17.50), 47	232.50 (NR), 100	232.80 (NR), 47				
BMC (Lumbar Spine)	364	Fritz. 2016c									83.80 (20.50), 172	81.30 (17.90), 92	236.70 (NR), 172	230.40 (NR), 92
BMC (Lumbar Spine)	416	Coster. 2016	82.60 (19.30), 65	77.70 (17.40), 39	266.30 (50.60), 65	245.5 (50.80), 39	85.60 (21.60), 93	86.70 (17.30), 37	261.00, 69.60), 93	267.50 (65.10), 37				
Bone Area (Whole Body)	32	McKay. 2005									1232.00 (178.00), 51	1228.00 (153.00), 73	1308.70 (NR), 51	1323.60 (NR), 73
Bone Area (Femoral Neck)	32	McKay. 2005									4.00 (0.40), 51	4.00 (0.30), 73	4.15 (NR), 51	4.16 (NR), 73
Bone Area (Greater trochanter)	32	McKay. 2005									5.30 (1.20), 51	5.10 (1.10), 73	6.06 (NR), 51	5.83 (NR), 73
Bone Area (Inter-trochanteric)	32	McKay. 2005									13.95 (1.90), 51	13.60 (1.60), 73	15.15 (NR), 51	14.50 (NR), 73
Bone Area (Femur)	32	McKay. 2005									23.30 (3.00), 51	22.70 (2.60), 73	25.40 (NR), 51	24.50 (NR), 73
Bone Area (Lumbar Spine)	32	McKay. 2005									39.20 (4.50), 51	38.50 (4.40), 73	41.50 (NR), 51	40.90 (NR), 73
Bone Area (Forearm)	156	Hasselstrom. 2008	6.28 (0.71), 135	6.31 (0.72), 76	7.42 (0.82), 135	7.29 (0.84), 76	6.66 (0.68), 135	6.78 (0.59), 62	7.49 (0.82), 135	7.58 (0.84), 62				
Bone Area (Calcaneus)	156	Hasselstrom. 2008	2.44 (0.64), 135	2.43 (0.54), 76	3.08 (0.44), 135	3.11 (0.43), 76	2.38 (0.67), 135	2.53 (0.61), 62	3.38 (0.40), 135	3.38 (0.44), 62				
CSA (Femoral Neck)	28	Fuchs. 2001									2.99 (0.08), 45	2.89 (0.06), 44	3.13 (0.08), 45	2.96 (0.07), 44
CSA (Femoral Neck)	52	Alwis. 2008					1.02 (0.2), 80	1.0 (0.17), 57	1.10 (NR), 73	1.09 (NR), 48				
CSA (Femoral Neck)	416	Coster. 2016	3.50 (0.40), 65	3.60 (0.50), 39	5.00 (0.40), 65	4.90 (0.60), 39	3.60 (0.40), 93	3.60 (0.30), 37	5.40 (0.50), 93	5.40 (0.60), 37				
CSA (Lumbar Spine)	28	Fuchs. 2001									36.43 (0.67), 45	37.25 (0.68), 44	38.44 (0.68), 45	38.91 (0.66), 44
CSA (Lumbar Spine)	416	Coster. 2016	27.50 (3.20), 65	27.90 (3.60), 39	50.00 (5.20), 65	48.30 (5.50), 39	29.10 (3.50), 93	29.90 (3.40), 37	54.50 (7.80), 93	55.50 (7.10), 37				
Width (L3)	104	Linden. 2006	2.86 (0.24), 49	2.91 (0.27), 50	3.14 (0.28), 49	3.08 (0.26), 50								
Width (Femoral Neck)	104	Linden. 2006	2.43 (0.24), 49	2.48 (0.32), 50	2.76 (0.32), 49	2.67 (0.27), 50								
aBMD (Whole Body)	32	McKay. 2005									0.78 (0.01), 63	0.81 (0.01), 81	0.81 (0.01), 63	0.82 (0.01), 81
aBMD (Whole Body)	36	Nogueira. 2014	0.71 (0.06), 12	0.73 (0.02), 6	0.76 (0.08), 12	0.74 (0.04), 6								
aBMD (Whole Body)	40	Barbeau. 2007	0.89 (0.07), 118	0.88 (0.06), 83	0.94 (0.08), 118	0.92 (0.07), 83								
aBMD (Whole Body)	104	Linden. 2006	0.84 (0.04), 49	0.84 (0.05), 50	0.90 (0.05), 49	0.89 (0.06), 50								
aBMD (Whole Body)	156	Meyer. 2013									0.65 (0.10), 149	0.65 (0.11), 65	0.79 (0.13), 149	0.78 (0.13), 65
aBMD (Whole Body)	364	Fritz. 2016b	0.68 (0.05), 72	0.68 (0.05), 45	0.98 (NR), 72	0.95 (NR), 45	0.69 (0.05), 100	0.70 (0.06), 47	0.98 (NR), 100	0.99 (NR), 47				
aBMD (Whole Body)	416	Coster. 2016	0.68 (0.05), 65	0.68 (0.05), 39	1.02 (0.08), 65	0.98 (0.09), 39	0.69 (0.05), 93	0.7 (0.05), 37	1.03 (0.11), 93	1.05 (0.1), 37				
aBMD (Forearm)	156	Hasselstrom. 2008	0.28 (0.03), 135	0.28 (0.03), 76	0.29 (0.03), 135	0.29 (0.04), 76	0.29 (0.03), 135	0.29 (0.03), 62	0.31 (0.04), 135	0.31 (0.04), 62				
aBMD (Calcaneus)	156	Hasselstrom. 2008	0.32 (0.05), 135	0.32 (0.05), 76	0.41 (0.06), 135	0.41 (0.06), 76	0.32 (0.04), 135	0.31 (0.04), 62	0.41 (0.05), 135	0.4 (0.06), 62				
aBMD (Leg)	104	Linden. 2006	0.76 (0.06), 49	0.76 (0.07), 50	0.88 (0.08), 49	0.85 (0.09), 50								
aBMD (Femur)	32	McKay. 2005									0.63 (0.01), 63	0.66 (0.01), 81	0.66 (0.01), 63	0.68 (0.01), 81
aBMD (Greater Trochanter)	32	McKay. 2005									0.52 (0.01), 63	0.53 (0.01), 81	0.54 (0.01), 63	0.55 (0.01), 81
aBMD (Hip)	156	Meyer									0.68 (0.09), 149	0.69 (0.10), 65	0.83 (0.15), 149	0.83 (0.14), 65
aBMD (Femoral Neck)	28	Fuchs. 2001									0.61 (0.01), 45	0.62 (0.01), 44	0.64 (0.01), 45	0.64 (0.01), 44
aBMD (Femoral Neck)	32	McKay. 2005									0.62 (0.01), 63	0.64 (0.01), 81	0.64 (0.01), 63	0.66 (0.01), 81
aBMD (Femoral Neck)	36	Nogueira. 2014	0.67 (0.08), 12	0.71 (0.06), 6	0.74 (0.10), 12	0.76 (0.08), 6								
aBMD (Femoral Neck)	104	Linden. 2006	0.72 (0.09), 49	0.71 (0.10), 50	0.80 (0.08), 49	0.79 (0.09), 50								
aBMD (Femoral Neck)	156	Meyer. 2013									0.65 (0.09), 149	0.66 (0.10), 65	0.78 (0.12), 149	0.78 (0.13), 65
aBMD (Femoral Neck)	364	Fritz. 2016b	0.71 (0.09), 72	0.70 (0.09), 45	1.01 (NR), 72	0.96 (NR), 45	0.78 (0.11), 100	0.78 (0.12), 47	1.00 (NR), 100	1.01 (NR), 47				
aBMD (Femoral Neck)	364	Fritz. 2016c									0.75 (0.10), 172	0.74 (0.11), 92	1.00 (NR), 172	0.99 (NR), 92
aBMD (Femoral Neck)	416	Coster. 2016	0.71 (0.1), 65	0.70 (0.09), 39	1.04 (0.13), 65	0.98 (0.14), 39	0.78 (0.11), 93	0.79 (0.12), 37	1.03 (0.14), 93	1.07 (0.15), 37				
aBMD (L3)	104	Linden. 2006	0.70 (0.08), 49	0.71 (0.08), 50	0.77 (0.09), 49	0.76 (0.09), 50								
aBMD (Lumbar Spine)	32	McKay. 2005									0.57 (0.01), 63	0.58 (0.01)	0.58 (0.01), 63	0.59 (0.01), 81
aBMD (Lumbar Spine)	36	Nogueira. 2014	0.65 (0.09), 12	0.69 (0.07), 6	0.72 (0.12), 12	0.73 (0.06), 6								
aBMD (Lumbar Spine)	156	Meyer									0.59 (0.10), 149	0.60 (0.11), 65	0.78 (0.16), 149	0.77 (0.17), 65
aBMD (Total Spine)	28	Fuchs. 2001									0.55 (0.01), 45	0.54 (0.01), 44	0.57 (0.01), 45	0.55 (0.01), 44
aBMD (Total Spine)	104	Linden. 2006	0.69 (0.08), 49	0.70 (0.08), 50	0.76 (0.09), 49	0.75 (0.08), 50								
aBMD (Total Spine)	364	Fritz. 2016c	0.68 (0.06), 72	0.69 (0.07), 45	0.99 (NR), 72	0.96 (NR), 45	0.68 (0.07), 100	0.69 (0.06), 47	0.93 (NR), 100	0.92 (NR), 47				
aBMD (Total Spine)	364	Fritz. 2016c									0.68 (0.06), 172	0.69 (0.06), 92	0.96 (NR), 172	0.94 (NR),92
aBMD (Total Spine)	416	Coster. 2016	0.68 (0.06), 65	0.69 (0.07), 39	1.05 (0.12), 65	0.99 (0.11), 39	0.68 (0.07), 93	0.70 (0.06), 37	0.98 (0.13), 93	1.00 (0.12), 37				
Legend: INT = Intervention group, CON = Control group, BMC = Bone mineral Content, L3 = Third Lumbar Vertebrae, CSA = Cross-sectional Area, aBMD = areal bone mineral density. NR = not reported. Notes: Data from Detter. 2013, Detter. 2014, Gutin. 1999, Gutin. 2008, Larsen. 2016, Lofgren. 2011, MacKelvie. 2004, Meyer. 2011, Specker. 2004 and Staiano. 2018 were not included as they did not provide raw follow-up values. Data from Fuchs. 2002 was not presented as it represents a subsample of Fuchs 2001 at the same time-point.														

## Appendix H. Peripheral Quantitative Computed Tomography

**Outcome**	**Timepoint (Weeks)**	**Study**	**Girls (Baseline)- Mean (SD), *n***		**Girls (Follow-up)- Mean (SD), *n***		**Boys (Baseline)- Mean (SD), *n***		**Boys (Follow-up)- Mean (SD), *n***		**All (Baseline)- Mean (SD), *n***		**All (Follow-up)- Mean (SD), *n***	
			**INT**	**CON**	**INT**	**CON**	**INT**	**CON**	**INT**	**CON**	**INT**	**CON**	**INT**	**CON**
vBMC (Tibia 4%)	36	Anliker. 2012									2.05 (0.45), 22	2.19 (0.37), 23	2.26 (0.52), 22	2.35 (0.35), 23
vBMC (Tibia 4%)	36	Nogueira. 2014	176.16 (28.16), 12	199.38 (19.19), 6	186.28 (21.67), 12	197.21 (19.76), 6								
vBMC (Tibia 14%)	36	Anliker. 2012									1.53 (0.35), 22	1.56 (0.20), 23	1.66 (0.36), 22	1.68 (0.24), 23
vBMC (Tibia 38%)	36	Anliker. 2012									2.10 (0.43), 22	2.19 (0.30), 23	2.27 (0.48), 22	2.35 (0.35), 23
vBMC (Tibia 66%)	36	Anliker. 2012									2.34 (0.49), 22	2.38 (0.49), 23	2.55 (0.53), 22	2.58 (0.41), 23
vBMC (Radius 4%)	36	Nogueira. 2014	41.20 (6.65), 12	48.40 (10.91), 6	52.66 (11.17), 12	50.41 (5.160), 6								
vBMD (Tibia 4%)	20	Meiring. 2014									304.60 (22.10), 12	319.60 (46.70), 10	306.20 (19.70), 12	306.20 (41.20), 10
vBMD (Tibia 4%)	36	Anliker. 2012									277.33 (28.89), 22	275.76 (25.78), 23	281.15 (28.52), 22	276.67 (24.01), 23
vBMD (Tibia 8%)	60	Macdonald. 2007	298.20 (36.40), 126	292.80 (32.30), 63	310.30 (41.40), 126	300.90 (34.60), 136	303.50 (30.40), 136	311.60 (32.20), 60	305.60 (29.90), 136	309.80 (34.00), 60				
vBMD (Tibia 14%)	36	Anliker. 2012									978.39 (23.97), 22	964.78 (24.70), 23	984.40 (23.97), 22	972.43 (28.16), 23
vBMD (Tibia 38%)	20	Meiring. 2014									1059.80 (51.40), 12	1071.10 (27.00), 10	1073.10 (44.40), 12	1071,10 (23.60), 10
vBMD (Tibia 38%)	36	Nogueira. 2014	1032.96 (34.67), 12	1046.33 (25.57), 6	1049.43 (25.99), 12	1049.30 (26.59), 6								
vBMD (Tibia 38%)	36	Anliker. 2012									1041.94 (29.04), 22	1032.58 (26.22), 23	1045.44 (28.93), 22	1038.60 (27.80), 23
vBMD (Tibia 50%)	60	Macdonald. 2007	1057.20 (33.70), 134	1058.60 (29.90), 64	1073.40 (35.80), 134	1067.00 (32.80), 64	1047.50 (35.80), 142	1046.00 (30.60), 64	1047.30 (35.70), 142	1046.00 (35.80), 64				
vBMD (Tibia 66%)	36	Anliker. 2012									1018.85 (22.48), 22	1006.00 (20.42), 23	1027.21 (24.82), 22	1012.78 (23.63), 23
vBMD (Tibia 66%)	208	Daly. 2016	1013.00 (34.00), 192	1016.00 (37.00), 170	1048.00 (NR), 94	1046.00 (NR), 69	1004.00 (36.00), 206	1015.00 (36.00), 159	1027.00 (NR), 97	1027.00 (NR), 76				
vBMD (Radius 38%)	36	Nogueira 2014	992.41 (42.25), 12	1004.74 (34.50), 6	1019.50 (32.61), 12	1008.04 (25.94), 6								
Trabecular BMD (Tibia 4%)	20	Meiring. 2014									270.20 (29.20), 12	291.40 (59.10), 10	277.20 (24.60), 12	264.10 (54.10), 10
Trabecular BMD (Tibia 4%)	36	Nogueira 2014	232.64 (26.05), 12	243.05 (10.15), 6	230.38 (26.63), 12	233.60 (10.74), 6								
Trabecular BMD (Radius 4%)	36	Nogueira 2014	218.32 (29.85), 12	219.56 (19.12), 6	230.23 (22.75), 12	224.08 (23.39), 6								
Total Bone CSA (Tibia 4%)	20	Meiring. 2014									802.00 (136.90), 12	738.50 (86.80), 10	847.80 (146.30), 12	741.30 (99.70), 10
Total Bone CSA (Tibia 4%)	36	Anliker. 2012									734.07 (128.55), 22	794.13 (126.25), 23	798.10 (137.15), 22	851.35 (115.98), 23
Total Bone CSA (Tibia 8%)	60	Macdonald. 2007	509.70 (74.70), 126	503.40 (80.00), 63	561.80 (77.60), 126	569.10 (90.90), 63	552.40 (88.20), 136	548.10 (88.10), 60	626.20 (100.60), 136	625.30 (94.70), 60				
Total Bone CSA (Tibia 14%)	36	Anliker. 2012									327.57 (64.04), 22	353.48 (51.90), 23	356.60 (66.71), 22	382.02 (52.85), 23
Total Bone CSA (Tibia 38%)	20	Meiring. 2014									294.90 (48.80), 12	269.20 (19.00), 10	304.10 (48.20), 12	279.40 (21.60), 10
Total Bone CSA (Tibia 38%)	36	Anliker. 2012									278.57 (56.89), 22	286.37 (38.35), 23	297.59 (57.45), 22	304.75 (43.72), 23
Total Bone CSA (Tibia 50%)	60	Macdonald. 2007	322.10 (51.60), 134	312.40 (53.70), 64	352.40 (54.20), 134	345.70 (56.90), 64	335.50 (54.00), 142	340.50 (49.90), 64	374.10 (62.10), 142	376.80 (58.60), 64				
Total Bone CSA (Tibia 66%)	36	Anliker. 2012									422.56 (98.46), 22	426.30 (53.77), 23	441.48 (102.22), 22	446.86 (61.95), 23
Total Bone CSA (Tibia 66%)	208	Daly. 2016	335.00 (51.00), 192	339.00 (58.00), 170	432.00 (NR), 94	459.00 (NR), 69	354.00 (61.00), 206	343.00 (58.00), 159	440.00 (NR), 97	450.00 (NR), 76				
Total Cortical Area (Tibia 38%)	20	Meiring. 2014									165.70 (26.10), 12	160.90 (17.50), 10	170.20 (25.00), 12	170.10 (17.20), 10
Total Cortical Area (Tibia 50%)	60	Macdonald. 2007	211.00 (33.10), 134	205.20 (36.50), 64	236.50 (37.50), 134	232.20 (41.60), 64	219.10 (36.00), 142	221.40 (30.60), 64	249.30 (42.80), 142	249.10 (36.60), 64				
Total Cortical Area (Tibia 66%)	208	Daly. 2016	150.00 (20.00), 192	154.00 (21.00), 170	232.00 (NR), 94	228.00 (NR), 69	145.00 (22.00), 206	154.00 (22.00), 159	237.00 (NR), 97	232.00 (NR), 76				
Total Cortical Thickness (Tibia 38%)	20	Meiring. 2014									3.40 (0.20), 12	3.30 (0.10), 10	3.40 (0.30), 12	3.40 (0.10), 10
Total Cortical Thickness (Tibia 66%)	208	Daly. 2016	2.69 (0.37), 192	2.75 (0.33), 170	3.94 (NR), 94	3.75 (NR), 69	2.71 (0.41), 206	2.72 (0.41), 159	4.00 (NR), 97	3.83 (NR), 76				
SSIPol (Tibia 14%)	36	Anliker. 2012									781.82 (248.63), 22	827.60 (161.14), 23	892.35 (270.53), 22	933.32 (190.72), 23
SSIPol (Tibia 38%)	20	Meiring. 2014									840.90 (180.70), 12	761.60 (77.30), 10	888.70 (187.10), 12	808.50 (99.00), 10
SSIPol (Tibia 38%)	36	Anliker. 2012									819.54 (239.80), 22	859.78 (174.23), 23	921.72 (269.10), 22	950.26 (205.84), 23
SSIPol (Tibia 50%)	60	Macdonald. 2007	954.40 (218.80), 134	915.50 (221.30), 64	1124.30 (251.50), 134	1093.50 (264.50), 64	1006.10 (241.40), 142	1029.80 (211.60), 64	1204.20 (295.50), 142	1208.00 (250.40), 64				
SSIPol (Tibia 66%)	36	Anliker. 2012									1290.15 (421.74), 22	1282.57 (251.26), 23	1419.45 (481.47), 22	1417.32 (303.63), 23
SSIPol (Tibia 66%)	208	Daly. 2016	905.00 (170.00), 192	943.00 (204.00), 170	1454.00 (NR), 94	1543.00 (NR), 69	974.00 (210.00), 206	937.00 (200.00), 159	1481.00 (NR), 97	1495.00 (NR), 76				
Bone Strength Index (Tibia 4%)	20	Meiring. 2014									7503.60 (1753.10), 12	7685.40 (2470.60), 10	7978.20 (1688.10), 12	7095.60 (2270.70), 10
Bone Strength Index (Tibia 8%)	60	Macdonald. 2007	4562.30 (1178.70), 126	4351.20 (1136.00), 63	5470.20 (1524.40), 126	5193.20 (1383.60), 63	5087.50 (1074.20), 136	5322.70 (1136.70), 60	5855.50 (1254.50), 136	6005.40 (1290.10), 60				
Periosteal Circumference (Tibia 38%)	20	Meiring. 2014									59.40 (3.30), 12	59.60 (1.80), 10	60.70 (3.80), 12	60.20 (1.60), 10
Endosteal Circumference (Tibia 38%)	20	Meiring. 2014									38.30 (2.70), 12	38.80 (1.10), 10	39.10 (3.00), 12	39.20 (1.00), 10

## Appendix M. Fracture Incidence within the Malmo POP Trial

**Timepoint (Years)**	**Study**	**Girls (INT)**		**Girls (CONT)**		**Boys (INT)**		**Boys (CONT)**		**All (INT)**		**All (CONT)**	
		**Fractures (*n*)**	**Sample (*n*)**	**Fractures (*n*)**	**Sample (*n*)**	**Fractures (*n*)**	**Sample (*n*)**	**Fractures (*n*)**	**Sample (*n*)**	**Fractures (*n*)**	**Sample (*n*)**	**Fractures (*n*)**	**Sample (*n*)**
3	Lofgren. 2011	12	362	33	780	25	446	38	807				
5	Detter. 2013	23	362	54	780	40	446	71	807				
6	Detter. 2014	33	417	65	835	53	500	98	869				
7	Fritz. 2016c									160	1339	281	2195

## Appendix N. Risk of Bias Assessment

**Study**	**Risk of Bias Arising from the Randomisation Process**	**Risk of Bias Arising from the Timing of Identification or Recruitment of Participants in a Cluster-Randomised Trial**	**Risk of Bias Due to Deviations from the Intended Interventions (Effect of Assignment to Intervention)**	**Risk of Bias Due to Deviations from the Intended Interventions (Effect of Adhering to Intervention)**	**Risk of Bias Due to Missing Outcome Data**	**Risk of Bias in Measurement of the Outcome**	**Risk of Bias in Selection of the Reported Result**	**Overall Risk of Bias**
Alwis. 2008	High	Low	High	High	Low	Low	Low	High
Anliker. 2012	Low	Low	High	High	Low	Low	Low	High
Barbeau. 2007	Low	Low	High	High	Low	Low	Low	High
Daly. 2016	Low	Low	High	Low	Low	Low	Low	High
Fuchs. 2001	Low	Low	High	High	Low	Low	Low	High
Gutin. 2008	Low	Low	High	High	Low	Low	Low	High
Hasselstrom. 2008	High	Low	High	High	Low	Low	Low	High
Larsen. 2016	Low	Low	High	High	Low	Low	Low	High
Macdonald. 2007	Low	Low	High	High	Low	Low	Low	High
MacKelvie. 2001	Low	Low	High	High	Low	Low	Low	High
McKay. 2000	Low	Low	High	High	Low	Low	Low	High
Meiring. 2014	Low	Low	High	High	Low	Low	Low	High
Meyer. 2011	Low	Low	High	High	Low	Low	Low	High
Nogueira. 2014	Low	Low	High	High	High	Low	Low	High
Specker. 2004	Low	Low	High	High	Low	Low	Low	High
Staiano. 2018	Low	Low	High	High	Low	Low	Low	High
Legend: INT = Intervention group, CON = Control group, vBMC = Volumetric bone mineral Content, CSA = Cross-sectional Area, vBMD = volumetric bone mineral, density, SSIPol = polar stress strain index.								
